# Surgical Management of Autosomal Dominant Polycystic Kidney Disease: Principles and Current Practice

**DOI:** 10.31729/jnma.8159

**Published:** 2023-05-31

**Authors:** Badri Man Shrestha

**Affiliations:** 1 Sheffield Kidney Institute, Sheffield Teaching Hospitals NHS Trust, Sheffield, United Kingdom

**Keywords:** *polycystic kidney disease*, *nephrectomy*, *transplantation*

## Abstract

Autosomal dominant polycystic kidney disease is the third most common cause of renal failure with no definitive treatment available that can directly target the development and growth of the cysts. Endeavours are being made to retard the growth of the cysts and preservation of renal function through medical treatment. However, 50% of the autosomal dominant polycystic kidney disease-affected persons develop complications and end-stage renal disease by the age of fifty-five and need surgical intervention for the management of complications, creation of dialysis access and renal transplantation. This review highlights the principles and current practice pertinent to the surgical management of autosomal dominant polycystic kidney disease.

## INTRODUCTION

Autosomal dominant polycystic kidney disease (ADPKD) is one of the most common congenital disorders affecting the kidney and the third leading cause of end-stage renal disease (ESRD) requiring renal replacement therapy (RRT). The natural course of ADPKD is characterized by a slow progression of cyst growth, destruction of renal parenchyma and deterioration of renal function leading to ESRD.^1^ There is no treatment until now that can directly target the mechanisms responsible for the development and growth of cysts in ADPKD, however initial medical management is directed mainly towards slowing down the progression of the disease.^2^ Surgical intervention is often required for the management of complications, and in preparation for RRT, that is, dialysis and renal transplantation (RT). The aim of this review is to discuss the role of surgery and the principles underlying the current surgical management of ADPKD.

## EPIDEMIOLOGY

There are over 10 million people worldwide affected by ADPKD and accounts for 5-10% of ESRD cases in USA and Europe. Fifty percent of people affected by ADPKD develop ESRD by the age of 55, which requires RRT.^3^ In Mayo Clinic, USA, over a period of 20 years (1984 - 2014), 472 out of 4213 (11%) RT recipients had ADPKD and 114/472 (24.3%) needed pre- or posttransplant native nephrectomy.^4^

## MANAGEMENT OF ADPKD

The manifestations of ADPKD are shown (Table 1).

**Table 1:** Manifestations of ADPKD.

**Renal cysts** **Extra-renal manifestations** Cysts in the liver, pancreas, seminal vesicles and arachnoid membranes Vascular malformations Intracranial aneurysms, thoracic aneurysm dissection and coronary artery aneurysms Cardiac manifestation Mitral valve prolapse and mitral regurgitation Colonic diverticulosis Abdominal wall hernias (Incisional, inguinal and paraumbilical hernias)

**Figure 1:**
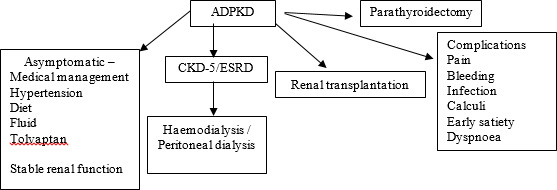
Pathway of management of ADPKD.

Because of multisystem involvement, multidisciplinary team management of ADPKD patients by nephrologists, radiologists and surgeons (kidney transplant, hepatobiliary, neuro- and cardiac surgeons) leads to the best outcome.^5-8^ All ADPKD patients are managed initially by nephrologists focussing on control of hypertension, fluid and electrolyte balance and continuous monitoring of renal function. Tolvaptan, a vasopressin-2 receptor antagonist, in multicentre randomised controlled trials, has been shown to slow the decline of renal function in patients with ADPKD at risk of rapid progression.^9^ The flow diagram shows the pathway of management of ADPKD at various stages of the evolution of the disease (Figure 1).

Measurement of total kidney volume (TKV) to monitor the burden of the disease and response to treatment is being done mainly in research settings. An important component of the initial management of ADPKD is to provide relevant information to the patients and their family members on the natural history of the disease, management issues, complications and eventual requirement of RTT in the form of dialysis or RT. The support given to the patients through dedicated ADPKD organisations contributes significantly towards overall management.^1,10^'^12^ Referral to a surgeon should be made for the management of complications refractory to conservative treatment, creation of HD or PD access, RT assessment and listing for RT and parathyroidectomy.^11^

## MANAGEMENT OF COMPLICATIONS

### Bleeding

Bleeding can occur within the cyst and pelvicalyceal system presenting as painful haematuria, which can also be a manifestation of renal calculi. A retroperitoneal or intraperitoneal bleed is associated with severe abdominal pain and features of hypovolaemia and anaemia.^13^ Majority of bleeding settles down on conservative treatment with rest, analgesics, blood transfusion and tranexamic acid infusion.^14^ Trans-arterial embolization of bleeding vessels successfully controls the bleeding in the cyst.^15^ Nephrectomy is indicated in patients with recurrent and intractable bleeding not responding to conservative treatment

### Cyst infection

Infection of the cysts is associated with pain and features of sepsis, which may require hospitalisation for treatment. Confirmation of diagnosis of an infected cyst may be challenging. Positron emission tomography may show abnormal uptake of fluorodeoxyglucose in the infected cyst.^16^ Rest, analgesics, fluid therapy and parenteral antibiotics administration (fluoroquinolones) resolve the infection in the majority of patients. Repeated hospitalisation from recurrent cyst infections is an indication for nephrectomy, particularly in patients who are waiting to have an RT because of the risk of life-threatening sepsis post-transplantation in the setting of immunosuppression.^17,18^

### Symptoms related to size

Symptoms such as abdominal pain, bloating, and early satiety may be related to the large size of the kidney compressing the stomach and intestine. Splinting of the diaphragm may be associated with dyspnoea on exertion and lying flat. Several procedures have been described to reduce the size of the enlarged kidneys but with unpredictable outcomes. Ultrasound aspiration and ethanol sclerotherapy in easily accessible cysts can offer temporary relief of symptoms. Trans-arterial embolization is reported to be successful in reducing the size of the kidneys by 36.3% at 3 months to 49% at 6 months.^19^ Laparoscopic decortication of the cysts and renal denervation is a less invasive technique which may provide relief of symptoms.^20^ In patients with compromised quality of life (QoL) from mechanical effects of the cyst, nephrectomy is indicated.^21^

## NEPHRECTOMY

**Figure 2:**
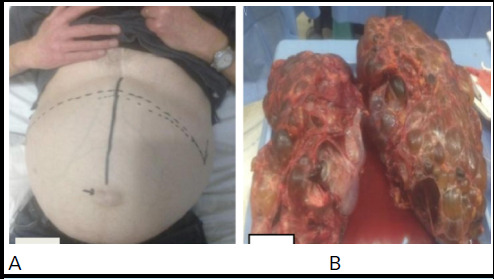
A) Bilateral subcostal rooftop incision marked prior to surgery, B) Polycystic kidneys.

The indications for nephrectomy include compromised QoL, pain, bleeding, recurrent cyst infection and for creation of space for an RT. The majority of nephrectomies are performed by open transperitoneal approach due to the large size of the kidneys. An anterior subcostal incision (unilateral or bilateral (rooftop)) provides satisfactory access to the enlarged kidneys (Figure 2).^22^

Identification and preservation adrenal gland should be performed to avoid inadvertent removal or devascularisation of the gland, which can lead to chronic adrenal insufficiency requiring hormonal replacement. Ligation of renal vessels at the hilum prior to mobilisation of the kidney helps reduce blood loss and the need for blood transfusion with the risk of allosensitisation. However, in some centres, polycystic kidneys are routinely removed laparoscopically with the added advantage of reduced post-operative pain, shorter hospitalisation and accelerated recovery.^23^

Simultaneous unilateral or bilateral native nephrectomy during the RT is being performed in some centres with no extra morbidity and mortality, which is done to reduce two separate operations and hospital stays.^24,25^ Mayo Clinic in the USA has published a classification based on imaging criteria, TKV adjusted for height (ht/TKV), which may be helpful in determining the need for nephrectomy pre-transplantation.^26^ However, there is no professional consensus regarding the timing (pre-/post-transplant), approach (open vs. laparoscopic), unilateral vs. bilateral and simultaneous nephrectomy and RT.^4^ Bilateral nephrectomy renders the patient anephric and anuric which impacts the fluid and nutrition management of the patient, and this needs to discussed with the patient prior to nephrectomy. The complications related to nephrectomy are listed in (Table 2).^27,28^

**Table 2:** Complications of nephrectomy.

Bleeding requiring blood transfusion with risk of sensitisation Adrenal insufficiency due to inadvertent removal of adrenal glands Paralytic ileus Injury to abdominal viscera Incisional hernias Neuralgic pain Anaemia related to decreased erythropoietin Vitamin D deficiency Acute kidney injury in the kidney transplant

## SIZE OF PKD AND PLD AFTER RT

In a published Japanese series, PKD size reduced by 37.7% at 1 year and 40.6% at 3 years, whereas liver size increased by 8.6% at 1 year and 21.4% at 3 years after RT.29 Similar observation was made in another study, therefore, caution should be taken while considering nephrectomy for size-related issues.^30^ On many occasions, the symptoms arising from PLD may be mistaken for those from PKD.

## RENAL TRANSPLANTATION

Because of the advantages such as improved quality of life, survival and cost-effectiveness, RT is the best form of RRT for patients with ADPKD. Pre-emptive RT, that is, RT before initiation of dialysis, confers better graft and patient survival. Therefore, planning RT well in advance, preferably with a timely living kidney donor (LKD) work-up offers the best opportunity for the avoidance of dialysis, which does carry significant morbidity and morbidity commensurate with the duration of dialysis.^31^ The surgical technique of RT, immunosuppressive drugs and the outcomes of RT in ADPKD patients are similar to those of non-ADPKD patients.^32^ An early referral by the nephrology team for RT assessment when the estimated glomerular filtration rate (eGFR) is between 15-20 ml/min/1.73 m^2^ gives plenty of time for undertaking an LKD work-up or creation of dialysis access.^33^

## ASSESSMENT FOR RT

A thorough clinical history-taking, physical examination and relevant investigations are necessary to establish the suitability of the patient for an RT (Table 3).

**Table 3:** Clinical assessment for RT in ADPKD.

**General risk assessment** **Specific assessment for ADPKD** Abdominal pain Back pain Early satiety Abdominal fullness Bloating Gastro-oesophageal reflux Dyspnoea Infection Haemorrhage (painful or painless gross haematuria) Hernias Headache Bowel symptoms

A clinical history of recurrent infection of the cysts, pain and bleeding into the cysts and urinary tract is important as nephrectomy may be needed prior to RT. A family history of intracranial aneurysm (ICA) or persistent headache mandates investigation with a magnetic resonance angiography (MRA) to exclude associated ICA, which, if present should be treated prior to RT to prevent a catastrophic bleed in the perioperative period.^34^

**Figure 3:**
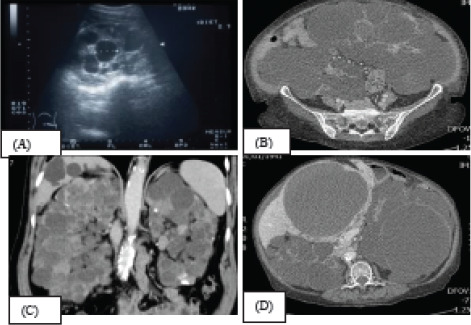
A) Ultrasound, CT, B) MR C) scan showing multiple renal cysts, and D) CT scan showing liver cysts.

Physical examination should be focussed specifically to assess the size of the polycystic kidneys and the availability of adequate space on the iliac fossae for implantation of the RT. A computerised tomographic (CT) or MR scan of the abdomen, depending on the eGFR, should be done to evaluate the adequacy of space in the iliac fossae if the clinical assessment is inconclusive (Figure 3).^35^

## ICA IN ADPKD

Undiagnosed ICA can lead to catastrophic intracerebral or subarachnoid bleeding in the perioperative period which can be associated with morbidity and mortality.^36^ In a series reported 12 of 105 ADPKD patients (12.4%) who had CT or MRI scans, demonstrated the presence of ICA. ICA, if present, should be treated by endovascular or surgical means prior to RT.^37^

## LIVING KIDNEY DONOR ASSESSMENT

**Figure 4:**
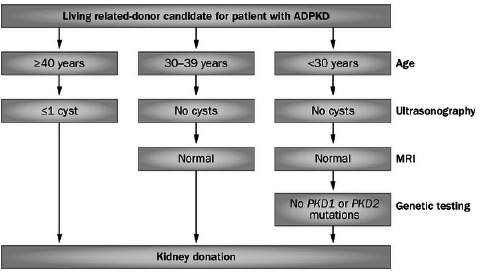
Showing the investigation criteria for LKD selection.

Living kidney donors coming forward from the same family as the recipients with ADPKD need special consideration because of the risk of transplanting a kidney with ADPKD and the risk of late manifestations of ADPKD in the donor.^38^ The indications for imaging and genetic testing for PKD^1^ and PKD^2^ mutations and diagnostic criteria for ADPKD while investigating an LKD from an ADPKD family, where cysts may not be detected in the early years of life, are outlined in the flow diagram (Figure 2 and 4).^39,40^

## DIALYSIS ACCESS

Both haemodialysis (HD) and peritoneal dialysis (PD) are possible in ADPKD patients and the choice between the two modalities depends on patients' personal preference and surgical suitability. In two published studies, no deleterious impact of kidney size on the outcomes on PD, such as overall patient survival and PD technique failure was observed. A large kidney size in patients with PKD is not a contraindication to PD and patients for whom a pre-transplant nephrectomy is mandatory can also safely opt for PD as a modality of dialysis.^41,42^ Recurrent cyst infection and flare of acute diverticulitis may predispose to recurrent peritonitis which may lead to switching to HD from PD.

In a large study comparing the dysfunction rates of arteriovenous fistula (AVF) and arteriovenous grafts (AVG) between ADPKD (n=557) and non-ADPKD (n=1671) patients on HD, ADPKD patients had lower incidence rates of AVF/AVG dysfunction within the first 180 days than non-ADPKD patients but presented a higher incidence rate after 1 year of AVF/AVG creation and onwards.^43^ ADPKD patients on HD tend to develop aneurysms more frequently, which is also related to the duration of dialysis vintage and usage of high-flux membranes with higher blood flow rate.^44^

## PLD WITH ADPKD

**Figure 5:**
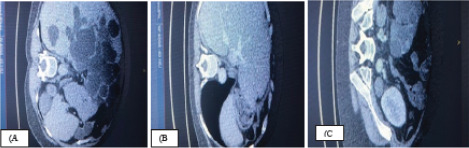
CT scan showing cysts before liver transplantation, B) after simultaneous liver and, C) renal transplantation (RT in the left iliac fossa).

75-90% of ADPKD patients have associated polycystic liver disease (PLD) which may remain asymptomatic or may develop symptoms due to its size and complications such as infection and haemorrhage.^45^ Symptomatic cysts can be managed by aspiration, sclerotherapy, percutaneous trans-arterial embolization, fenestration, resection and liver transplantation (LT).^46^ In patients with end-stage ADPKD and PLD, simultaneous or sequential RT and LT are being performed successfully with excellent outcomes (Figure 5).^47,48^

## PARATHYROIDECTOMY

The parathyroid glands on the neck are responsible for monitoring and regulating blood calcium levels. Due to an imbalance of calcium, phosphorus and vitamin D levels in renal failure, parathyroid glands become overactive, causing osteoporosis, bone pain and fractures. Persistent hyperparathyroidism posttransplantation, which can be identified in up to 80% of transplant recipients, is associated with adverse graft and patient outcomes, including higher fracture risk and an increased risk of all-cause mortality and allograft loss.^49^ Medical management including vitamin D, phosphate binders and calcimimetic agent (Cinacalcet) is successful in controlling hyperparathyroidism in the majority of patients. Parathyroidectomy is indicated in intractable cases of secondary and tertiary hyperparathyroidism.^50^

## WAY FORWARD

ADPKD is the third most common cause of ESRD and a multidisciplinary team approach is essential for the long-term preservation of renal function. The majority of ADPKD are managed medically initially, but surgical intervention is often required in half of these patients in long term. A timely referral for pre-emptive RT from an LKD provides the best outcomes. Support to ADPKD patients and their family through dedicated organisations have an important place in the overall management of ADPKD.
